# Overview of general surgery medical residency programs and prerequisite program in basic surgical area in Brazil: Historical review and update

**DOI:** 10.1590/0100-6991e-20223410_en

**Published:** 2022-11-10

**Authors:** ELIZABETH GOMES DOS SANTOS, VIVIANE CRISTINA ULIANA PETERLE, MAGALI MACHADO SANCHES, ADNAN NESER, MARCELO DI BONIFACIO, LUIZ CARLOS VON BAHTEN, FERNANDO TALLO, MAURO RIBEIRO, ROSANA LEITE DE MELO, PAULO ROBERTO GOMES DOS CORSI

**Affiliations:** 1 - Comissão Nacional de Residência Médica - Rio de Janeiro, RJ - Brasil; 2 - Comissão Nacional de Residência Médica - Brasília - DF - Brasil; 3 - Comissão Nacional de Residência Médica - São Paulo - SP - Brasil; 4 - Comissão Nacional de Residência Médica - Ribeirão Preto - SP - Brasil; 5 - Colégio Brasileiro de Cirurgiões - Curitiba - PR - Brasil; 6 - Associação Médica Brasileira - São Paulo - SP - Brasil; 7 - Conselho Federal de Medicina - São Paulo - SP - Brasil

**Keywords:** Education, Medical, General Surgery, Internship and Residency, Health Services, Residência Médica, Educação Médica, Cirurgia Geral, Área Básica, Sistemas e Serviços de Saúde

## Abstract

**Objective:**

to describe vacancy regulation process by adding, describing the panorama of the General Surgery Residency Program (PRMCG) and the Basic Surgical Prerequisites Program (PRACB).

**Method:**

descriptive, quali-quantitative, cross sectional study conducted from document analysis from National Commission of Medical Residency (CNRM).

**Results:**

in 2018, after evaluation of the General Surgery Services for adequacy of the number of vacancies (DS), the PRACB was instituted as a modality of access to surgical specialties until definitive change in the time of the formation of the general surgeon for three years, in 2022. In the first addition of vacancies in 2018, 127 PRMCG were authorized with 736 vacancies of R1 and 290 PRACB (2 years) with 1.286 vacancies offered for R1. In the second addition in 2021, 423 PRM were authorized with 1.564 R1 vacancies in PRMCG.

**Discussion:**

the regulation of the offer of vacancies for the formation of specialties in Brazil should align the evaluation of practice scenarios with the profile of skills. The PRACB modality was instituted for a certain time for budgetary preparation and practice scenarios until the complete transition to training in 3 years.

**Conclusion:**

Brazil by 2018 was the only country to grant the Board Certification to General Surgeon with only 2 years of training. After a transitional period the same analysis methodology for adding and regulating vacancies was applied to services.

## RESUMO

**Objetivo:**

descrever o contexto do processo de construção e implantação da Matriz de Competências da Cirurgia Geral com tempo de formação em 3 anos e o processo sobre a regulação de vagas, por aditamento, descrevendo o panorama dos Programas de Residência Médica em Cirurgia Geral (PRMCG) e do Programa de Pré-Requisito em área cirúrgica básica (PRACB).

**Método:**

estudo descritivo, quanti-qualitativo, transversal, realizado a partir da análise documental das atas, súmulas, nota técnica, resoluções e atos autorizativos da Comissão Nacional de Residência Médica (CNRM), bem como do Diagnóstico Situacional (DS), considerando a matriz de competências da especialidade em sua implementação, por meio de dados coletados no Sistema da Comissão Nacional de Residência Médica (SisCNRM), no portal eletrônico do Ministério da Educação (MEC).

**Resultado:**

em 2018, após metodologia de avaliação dos serviços para adequação do número de vagas (DS), foi instituído o PRACB como uma modalidade de acesso às especialidades cirúrgicas e de adaptação dos serviços formadores, até mudança definitiva no tempo de formação do Cirurgião Geral, para três anos, em 2022. No primeiro aditamento de vagas, em 2018, foram autorizados 127 PRMCG (3 anos) com 736 vagas de R1 e 290 pracb (2 anos) com 1286 vagas ofertadas para R1. No segundo aditamento, em 2021, foram autorizados 423 PRM com 1564 vagas de R1 em PRMCG. Atualmente 2495 médicos residentes estão em curso nos PRMCG e 50 (R2) estão concluindo o pracb. A maior concentração de PRM e médicos residentes encontra-se no Estado de São Paulo, em oposição a Região Norte.

**Discussão:**

a regulação da oferta de vagas para a formação de especialistas no Brasil deve alinhar a avaliação dos cenários de prática ao perfil das competências para a Especialidade como as necessidades de médicos especialistas indicada pelo perfil socioepidemiológico da população. Contudo, as instituições devem possuir estrutura compatível para a aquisição de competências necessárias ao especialista e dotação orçamentária para o financiamento do processo. A modalidade PRACB foi instituída por tempo determinado para a preparação orçamentária e dos cenários de prática, até a transição completa para a formação em 3 anos, possibilitando ao médico residente, enquanto vigente, acesso às especialidades cirúrgicas.

**Conclusão:**

O Brasil até 2018 era o único país a conceder o Título de Especialista em Cirurgia Geral com um tempo exíguo de treinamento - 2 anos. Após um período de transição, a mesma metodologia de análise para aditamento e regulação da oferta de vagas foi aplicada aos serviços. A partir de 2023, o PRMCG com duração de 3 anos, será a modalidade de formação que confere o título de especialista em Cirurgião Geral pela CNRM, promovendo qualificação profissional para assistência e cenários de prática compatíveis com a aquisição de competências. .

## INTRODUCTION

Medical Residency (MR) is a course for training specialists in areas of medical knowledge, recognized worldwide as the best form of training after graduation^1^. The objective of a good General Surgery Medical Residency Program (GSMRP) is to train a surgeon to work with autonomy, professionalism, ethics, and great technical skills^1^.

MR was instituted in Brazil by presidential decree in 1977 and defined as “a postgraduate teaching modality for doctors, characterized in the form of a specialization course with in-service training, working in health institutions, university or not, under supervision of technically and ethically qualified professionals”. It is governed by specific laws and regulations^2^.

For regulatory purposes, the National Commission for Medical Residency (Comissão Nacional de Residência Médica - CNRM) was created that same year, as a collegiate instance directly linked to the federal government via the Ministry of Education, whose functions are the organization, accreditation, and monitoring of residency programs distributed over Brazil^3^.

Since 2005, the Brazilian College of Surgeons (CBC) has worked together with the CNRM, the Brazilian Medical Association (AMB), the Federal Council of Medicine (CFM), and other specialty societies, with discussions throughout the national territory, to adapt a new General Surgery Matrix to the General Surgery program. 

In 2018, CNRM carried out the first evaluation of the GSMRPs to adjust the number of vacancies to be offered. At this time, the Prerequisite Program in Basic Surgical Area (PRACB) was instituted as a transition modality for the purpose of adapting training services until the definitive change in the training period of the General Surgeon to three years in 2022^4^.

The regulation of the offer of vacancies for the training of specialists in Brazil must consider the practice scenarios in relation to the requirements for the Specialty, as well as the needs of specialist physicians indicated by the socio-epidemiological profile of the population. In 2021, a new evaluation of the programs was necessary to verify the installed training capacity in the practice scenarios of the GS and PBSA programs^5^.

Currently, there are Residency Programs authorized in 55 medical specialties and in 59 practice areas recognized by the CFM^6^. The Joint Specialties Commission, constituted by CNRM, AMB, and CFM, updated the list of Medical Specialties in 2018, with the inclusion of increased training time for General Surgery, in line with the publication by CNRM of the three-year Competence Matrix (CM) for the General Surgery Program^6^.

## GOAL

To describe the national panorama of GS and PBSA, pointing out the distribution of vacancies by Brazilian states and the number of residents, in addition to a historical review of the implementation and extinction of PBSA, from 2018 to 2021.

## METHODS

This is a descriptive, cross-sectional study, carried out from data collected in the National Commission of Medical Residency System (SisCNRM), through the Ministry of Education (MEC) electronic portal, extracted between March 1^st^, 2021 to May 30^th^, 2021.

We selected the variables according to the number of residents attending GS and PBSA in 2019 and 2020, analyzing the total residents of the first (R1), second (R2), and third (R3) years of training, by state, for GS and of R1 and R2, by state, for PBSA. To calculate the number of vacancies, we included all programs accredited by Federation Unit, including programs that were listed as “approved”, “expired”, “diligence” and “requirement” in SisCNRM.

## RESULTS

### I - Historical review

The objective of the Medical Residency training model is to train specialist professionals with a high technical-scientific level to work in health care^1^. MR is a postgraduate course in the form of training in health services. While the professional develops skills in a certain area of knowledge under supervision, he/she also serves assisting the population. And by law, at the end of a successfully completed training, the professional is granted the Board Certification^1^.

At the time of MR creation in Brazil, the areas of knowledge covered were Internal Medicine, General Surgery, Pediatrics, Obstetrics and Gynecology, and Preventive and Social Medicine. The country needed specialists in these areas and the degree was also necessary for the admission of doctors with degrees in state and parastatal bodies^3^.

Such programs were intended for medical school graduates. Therefore, access was direct, through a selection process, without prerequisites. CNRM resolutions established that MR programs in these areas would have a duration of two years, and that for the new accredited surgical specialties, the prerequisite in General Surgery would be mandatory^3^.

Until CNRM Resolution No. 02/2006, the General Surgery program had direct access, lasted two years, and was a prerequisite for other surgical specialties^8^. This program, which lasted until 2018, consisted of nine rotations in other specialties lasting 30 days in the first year (Surgical Technique, Intensive Care, Pediatric Surgery, Plastic Surgery, Vascular Surgery, Thoracic Surgery, Head and Neck Surgery, Urology, and Coloproctology), one month of vacation each year, plus 11 months of rotation in General Surgery. The workload of 2,880 hours/year was divided into 25% in the ward, 15% in the outpatient clinic, 25% in the operating room, 15% in urgency and emergency services, and 10% theoretical activities^7^ ^,^ ^8^.

The Brazilian College of Surgeons (CBC) was founded on July 30^th^, 1929, by a group of surgeons who have long been committed to medical education in General Surgery (GS), mainly offering Congresses, Conferences, Seminars, among the various educational forms possible. Since then, it has been involved in the training of the general surgeon^9^. 

Technological developments and the incorporation of new knowledge in medicine have raised concerns about the training of the General Surgeon and about the integral acquisition of knowledge, and since 2004 CBC has been working together with CNRM to adapt a new GSMRP Matrix to the 21^st^ century, with discussions throughout the national territory. In September 2004, CBC took to CNRM a proposal for a change to a four-year duration that was not approved, but a period called “additional years” was approved for deepening knowledge, called Advanced Program in General Surgery, with two years duration, thus completing the four years proposed by CBC^10^ ^,^ ^11^.

The studies continued, and in 2010 CBC implemented a new project to change the formatting of GS. At the end of 2012, a national survey was carried out with all CBC’s Titular (TCBCs) and Emeritus (ECBCs) Members registered as general surgeons, aimed at knowing how many years should the GS residency program be and which rotations were considered the most relevant in the integral formation of the specialist. The result of the research was taken to the CBC National Directory in 2013 and approved; in July of the same year, it was taken for the first time to the CNRM plenary.

Most responses indicated three years as the minimum acceptable for the duration of the program, with an ideal of four, and some considered up to five years, like in the USA. The essential rotations mentioned were Urology, Coloproctology, Thoracic Surgery, and Vascular Surgery, each lasting three months^12^.

With these definitions for the proposed change, a new pedagogical matrix was built and presented to CNRM, which, after the necessary adjustments, was approved in November 2017.

Then, CFM held two discussion forums with the other surgical specialties to evaluate and plan the program change.

The first forum^13^ took place on March 21^st^, 2017, with the presence of several presidents from other surgical specialties, highlighting the importance of extending the time of the specialty. In the second forum^14^, on May 4^th^, 2018, it was defined that the GS would become a three-year program, with a new Competence Matrix (CM), now with defined objectives, competencies to be acquired, and operations to be carried out by year of training and granting the Title of Specialist in General Surgery at the end^15^.

On the same occasion, at the request of other surgical specialties that have the GSMRP as a prerequisite, and as a way of adapting services to the practice scenarios required by the new matrix and the preparation of managers regarding the funding of grants to resident physicians, an intermediate system creation was discussed, and the Prerequisite Program in Basic Surgical Area (PBSA) was then instituted, lasting two years, whose CM with regard to R1 and R2 would be the same as GS^15^.

Despite offering the cost of the scholarship to the resident physician, this program would not qualify him/her as a specialist in General Surgery, being only a prerequisite modality for selection processes for other specialties that for which General Surgery is a prerequisite. The prerequisite was instituted as a transitional one, to be reassessed in five years, a maximum period in which all programs would pass to three years^15^.

The CM for the three-year GS was definitively approved with the publication in the Federal Official Gazette, on December 14^th^, 2018, of the Resolution nº 48, of June 28^th^ of the same year^16^. As of 2019, it became mandatory for GS programs to last for three years, granting the Title of Specialist at the end of training, but there would be a transition interval.

The new CM was built based on the learning domains according to Bloom’s Taxonomy^16^: cognitive (knowledge), psychomotor (development of technical skills), and affective (attitudes and judgments), and assessment by Miller’s Pyramid^17^, where at the base there is cognitive learning, the acquisition of knowledge, the second stage is learning how to do it, in the third the apprentice demonstrates what she/he has learned, and in the fourth and final stage the resident actually performs in daily practice.

For the adequacy of the number of vacancies, it was necessary that each institution had an installed capacity to be accredited. CNRM carried out a detailed study entitled “Situational Diagnosis” with all GS services in the country^4^.

### II - Amendment and distribution of vacancies in the programs of general surgery residence and prerequisite in basic surgical area.

 In the second half of 2018, through a circular letter from CNRM, a questionnaire (DS) was sent to all State Medical Residency Commissions (CEREM), Residency Commission Coordinators (COREME), and Program Supervisors of all institutions that already provided GSMRP. The questionnaire aimed at assessing the surgical potential of the program^21^.

The questionnaire consisted of quantitative and qualitative variables regarding the competencies expected in the matrix for each year, according to the surgical procedures performed by each service. The analysis compared the number of vacancies requested in the Provisional Accreditation Program (PAP) and the information in the questionnaire with the documents proving the productivity of the service.

After evaluating the responses and analyzing the CEREMs in on-site visits for new programs, regulation was carried out and the operation of the GS program (three years) or the PBSA program (two years) or both were authorized, depending on the institution’s installed capacity, according to the distribution by Federation Unit. The authorized vacancies were made available for access through a public selection process as of 2019, configuring the annual panorama shown in Table 1. 

**Table 1:** National Distribution of GSMRP and PRPBSA, according to the number of vacancies offered, as of 2019.

Period	Year	Residents studying GS	Residents studying PBSA
R1	2019	736	1286
R1	2020	662	1355
R1	2021	423	1564

Source: SISCNRM/CTCG-CNRM.

As for the number of programs and residency vacancies held in 2018, 94 GSMRP were added, and 619 vacancies were opened. Eighteen programs offering 60 vacancies had provisional accreditation, those that never had GS, and 15 programs with 47 vacancies were accredited for five years, those that already had their process in progress, before the DS.

Overall, 736 vacancies were authorized for R1 for the 2019 selection processes, with a duration of three years, distributed in 127 Medical Residency Programs throughout Brazil.

Regarding PBSA, all programs were provisionally accredited, in a total of 290 MRPs, with 1,286 vacancies offered for R1. Of all the existing General Surgery programs, 96 chose to only offer vacancies as PBSA, most of them in the state of São Paulo^17^.

Regarding the geographical distribution, there was a predominance of programs in the Southeast region, with 116, corresponding to 48.1% of the total GSMRPs in Brazil. The discrepancy is clear compared with other regions, with 24.8% of vacancies in the South region, 17.4% in the Northeast, 8.2% in the Midwest, and 6.6% in the North^17^.

The Southeast region also has a great predominance of PRPBSA, 152, 48.5% of the total number of vacancies, especially in the state of São Paulo, where are located the programs with the highest number of vacancies and resident physicians in the system, 495 resident physicians, corresponding to 37.2% of the vacancies in Brazil^17^.

In the opposite direction the Federation Units with the lowest number of resident physicians attending GSMRP, with less than six residents, in 2019, were Amapá and Sergipe, with two residents, and Tocantins, with three. The lowest number of resident physicians attending the PRPBSA predominated in the North region in 2019, mainly in Amapá, followed by Acre, Rondônia, and Roraima^17^.

Training in General Surgery after the residency can continue with the three areas of expertise, Bariatric Surgery (two years), Trauma Surgery (one year), and Laparoscopy (one year), or with two additional years to deepen knowledge, the Advanced Program, currently with 11 active programs and 32 R1. All have matrices already written and in force.

### III - Amendment and distribution of vacancies in residence programs in general surgery and extinction of the prerequisite program in basic surgical area in 2022

#### A - COVID-19 Pandemic

On March 11^th^, 2020, the World Health Organization (WHO) declared that the infection with the new coronavirus, SARS-CoV-2, until then considered a Public Health Emergency of international importance, had reached the status of a pandemic, which forced a reorganization of health services to serve the population, subsequently modifying the practice scenarios for the execution of Medical Residency programs in Brazil^20^.

This new conformation of health services was reported by COREME as the main complicating factor for the execution of the MRP in its entirety, making it difficult to comply with the CM specific for each area.

The complexity of health care in the face of COVID-19 required a diversity of professionals and doctors in priority and specific areas, especially specialists focused on hospital care. Faced with this need, doctors in medical residency programs were relocated as a means of filling the lack of human resources to face the pandemic.

On the other hand, there has also been a progressive and drastic reduction in the demand for service provision in areas not directly related to COVID care, especially in elective, clinical, or surgical care, as well as diagnostic or therapeutic investigation, either due to social isolation, decreased demand for treatment for other pathologies, or the redistribution of resources and supplies within institutions.

Brazil being a continental country, the offers of assistance services and the quantity of material and human resources, usually display considerable regional variations. In the context of the pandemic and the different epidemiological situations of case incidence and lethality rates by each region/state, the differences in the execution of responses between the services were also clear.

In the meantime, CNRM met continuously in an extraordinary way, demonstrating that it closely followed the various and different demands, which culminated in actions such as the Technical Note for guidance for COREMES, to guide and reduce the effects of pressure from the health system on the training process^21^.

An extensive situational survey was also carried out among the institutions, to assess the pandemic local impact regarding training and expectations of extending the regular time of MRPs, an unprecedented situation in 44 years since Medical Residency was officially established in Brazil. Of the 10,753 resident physicians who participated in the survey, 68.7% were from surgical areas, and considered it possible to recover content during the MRP, without the need to extend the training period^4^.

#### B - Work Process - Termination of PBSA

In August 2020, at a CNRM Plenary Meeting, CBC put on the agenda the concern with the training of the General Surgeon in relation to the acquisition of the necessary skills for the integral care of the population and proposed the extinction of PBSA right after the conclusion of its first class, in 2020, presenting as a basis for the request the content of the resolutions and directives that created the GS specialty^22^ ^-^ ^25^.

The Plenary, however, considered that the extinction could not be carried out suddenly, due to the unexpected context of the pandemic and the need for monitoring the acquisition of skills. Due to the advancing time for the inclusion of authorizing acts, in 2020 PRPBSA could not be extinguished, the offer of vacancies being allowed, without number increase, in the Selection Process for 2021^4^.

One of the reasons that strengthened the need to terminate PRPBSA was the number of residents who dropped out of the program, as shown in Table 3. It was also found that more than 50% of the residents who completed were not approved for any other surgical specialty.

**Table 2:** Number of resident physicians attending PBSA and GS in the years 2019 to 2021.

Period	Residents attending PRPBSA	Residents completed PRPBSA	Residents attending GSMRP
	(R1)	(R2)	
2019	1286	-	736
2020	1355	-	662
2021	1326	1034	721
2022	50	1061	1623

Source: SisCNRM /CTCG-CNRM.

**Table 3:** Survey response on the possibility to recover training.

		Do you think it's possible to recover?			TOTAL
			NO	YES	
MRP AREA	CLINICAL	10	1.039	3.404	4.453
		0.2%	23.3%	76.4%	100.0%
	SURGICAL	25	1.076	2.532	3.633
		0.7%	29.6%	69.7%	100.0%
	URGENCY	2	24	135	161
		1.2%	14.9%	83.9%	100.0%
	OTHERS	7	159	416	582
			27.3%	71.5%	100.0%
TOTAL		44	2.298	6.487	8.829
		0.5%	26.0%	73.5%	100.0%

Source: National Commission of Medical Residency.

**Table 4:** Withdrawal from PRPBSA.

Period	PRPBSA withdrawal
2019	227 (18.30%)
2020	275 (20.29%)
2021	296 (22.32%)
2022	-

Source: CNRM.

**Table 5:** Distribution of residents in other surgical specialties in 2021.

Surgical Specialty	2021 (R1)
Vascular surgery	292
Urology	248
Coloproctology	161
Plastic surgery	179
Thoracic surgery	71
Pediatric surgery	89
Surgery of the Digestive System	157
TOTAL	1197

Source: CNRM/CTCG.

After plenary discussions, it was decided that the same procedure carried out when the PBSA was created was to take place again, with the individual reassessment of the services regarding the installed capacity for a new addition of vacancies to be offered, through a new, more extensive questionnaire that would quantitatively and qualitatively evaluate the service for the third year of the GS considering the competence matrix^4^.

For a gradual process, it was determined that requests for increased vacancies or five-year accreditation for PBSA (two years) were suspended, as well as requests for increased vacancies or five-year accreditation for GS at that time. The rules governing PBSA were then maintained, guaranteeing that upon completion of the program (two years) the resident would receive a Certificate of Acquisition of these competences relating to R1 and R2, as provided for in the resolution in force^26^.

In May, June, July, and August 2021, the processes for the new DS of the services took place together with all the Institutions to add vacancies for the GS (three years), the only training modality from 2022 on. Respecting the same methodology after analyzing the given the data, the institutions and the State Medical Residency Commissions were heard, to carry out a thorough analysis for the addition and regulation of vacancies in October 2021.

Data analysis showed 1,564 vacancies added to GSMRP for 2022, with a total of 423 programs. In relation to 2021, there was an increase of 53.9% vacancies (843)^17^.

### III - Proportionality of access to surgical specialties

There was again a great concern, which had already been discussed when the GS CM was approved for three years, that with the increase in the duration of the GSMRP there would be a decrease in access to the programs of the other surgical specialties. It was questioned whether there would be a decrease in vacancies for other specialties. CNRM then carried out a survey in its SisCNRM database and showed that there was no change in the number of vacancies offered for the other surgical specialties and there would be no decrease in the number of vacancies for the prerequisite.

## DISCUSSION

How much training time does a resident need to learn to perform operations autonomously? After how many operations can a surgeon be considered a specialist? These are undoubtedly open questions and have been the subject of a great deal of debate so far^27^.

What should the ideal program look like? Opinions vary. There are those who advocate that emphasis should be given to research, and others who believe intense training in virtual laboratories is essential. The common point for all authors is that the program is of sufficient duration so that it can offer high-quality training and all resident physicians are equally exposed to the essential elements for their future practice: performing enough operations, since without operating someone does not become a surgeon^28^ ^,^ ^29^.

Although the exact number of procedures required to acquire specialist status is not known, and although this varies among resident physicians, the consensus imposes the definition of in-service training, that is, only by practicing will surgeons be able to acquire competences, and in the most important practice scenario, the operating room. Except for the extremes of the learning curve, the average training of surgeons needs time and repetition of movements^29^ ^-^ ^31^. 

As of January 2020, considering the state of Public Health emergency caused by COVID-19^20^, CNRM made a great effort to mitigate the impact of the pandemic on MRPs in Brazil, trying to maintain their quality, especially in relation to programs in surgical areas that depend more on the practical part. Considering the impact on the elective procedures, necessary for the training process, the Commission prudently evaluated the changes in the existing training models, including the PBSA transition model, until better definition of the scenarios.

However, the questions about the PBSA program, an intermediate modality and not providing qualification as a specialist, offering only a Certificate of Acquisition of Skills, and the high dropout rate soon after its creation, made it necessary to rethink its validity, then emerging the idea of its closure. After many discussions, including with CBC, CFM, the March 2021 CNRM plenary session unanimously decided that vacancies for this program will no longer be offered for 2022. In addition, PBSA will terminate at the end of the 2020 class (February 2022)^4^. Importantly, the final PRPBSA vacancies purport to the reservation requests of residents destined for military service.

CNRM carried out new questionnaires, similar to the one previously used for the 2018 amendment with the institutions. The CNRM General Surgery Technical Chamber conducted an extensive analysis of the relationship between the Competency Matrix and the installed capacity of each program to authorize the number of vacancies for GS to be made available for the 2022 selection processes, the procedure regarding the regulation of medical residency vacancies. PBSA were meticulously evaluated to verify if they could offer vacancies for GS. Only 16 programs were not authorized to offer vacancies for GS^32^.

## CONCLUSION

The process to amend as well as to terminate PRPBSA took place after several studies, analyzes, and discussions. Data collected from SisCNRM showed that after the conclusion of the first class in February 2020, more than 50% of those graduating from this modality were not included in other surgical specialties, leaving many unqualified professionals in the labor market to exercise their profession as surgeons. There is still no data to support the hypotheses for this phenomenon. The most likely option is that, in addition to not being approved in the selection processes, a percentage that cannot be quantified chose not to attend another residency for the time being. From 2022 on, with the end of PBSA, Brazil will only have surgeons titled as specialists, even if they later enter other surgical areas.

### In time:

The professional situation of PBSA graduates greatly concerned the Tripartite Commission - CFM, AMB, and CNRM -, as well as CBC. The test for granting of the Specialist Title by the CBC/AMB association follows strict rules, such as the training period (six years). After meetings and discussions, at the request of CNRM and with the consent of the CBC, which demonstrated great accessibility and empathy towards the topic, it was decided that the CBC Specialist Title Committee will exceptionally accept the PBSA certificate of completion as one of the requirements for application to its selection^33^.

## References

[1] Santos EG (2015). "Avaliação da Habilidade Técnica na Residência Médica em Cirurgia Geral".

[2] MEC (1977). Decreto nº 80.821 de 5 de setembro de 1977.

[3] MEC (1981). Lei 6932 de 20 de janeiro de 1981.

[4] ATA (2020). CNRM de 27 de novembro de 2020.

[5] Junior Miranda (1997). UJP. Comissão Nacional de Residência Médica: Caminhos e descaminhos na gestão desta modalidade de especialização médica no brasil.

[6] CFM (2018). Resolução nº 2221 de 23 de novembro de 2018..

[7] MEC http://siscnrm.mec.gov.br/login/login.

[8] MEC (2006). Resolução 02 /2006 de 17 de maio de 2006..

[9] Colégio Brasileiro de Cirurgiões (2007). A arte e a técnica da cirurgia no Brasil.

[10] Saad R (2005). Editorial Boletim CBC.

[11] Rasslan S, Birolini D (2008). " Programa Avançado de Cirurgia Geral".

[12] CBC ATA da reunião do Diretório Nacional.

[13] CFM I Fórum de Cirurgia Geral.

[14] CFM II Fórum de Cirurgia Geral.

[15] MEC http://portal.mec.gov.br.

[16] Diário Oficial da União https://www.in.gov.br.

[17] SisCNRM (2022). SisCNRM.

[18] Domínios de Bloom http://formacao.fikaki.com/manual/definicao-objectivos-pedagogicos/dominios.

[19] Miller S Pirâmide de Miller.

[20] OPAS https://www.paho.org/pt/news/30-1-2020-who-declares-public-health-emergency-novel-coronavirus.

[21] MEC (2018). Ofício-Circular nº 55/2018/CGRS/DDES/SESU/SESU-MEC.

[22] (2018). Resolução n°4 de 28 de junho de 2018..

[23] (2018). Ofício circular CNRM n°89 35/2018..

[24] (2018). Processo CNRM 23000.020937/2018-34..

[25] (2018). Ofício Circular CNRM n° 55 e 56 /2018..

[26] (2021). Resolução CNRM n° 2 de 15 de março de 2021..

[27] Jackson GP, Tarpley J (2009). How long does it take to train a surgeon. BMJ.

[28] Lun SK, Crisostomo AC (2009). A comparative study of surgical training in South East Asia, Australia and The United Kingdom. Asian J Surg.

[29] Mattar SG, Alseidi AA, Jones DB (2013). General surgery residency inadequately prepares trainees for fellowship results of a survey of fellowship program directors. Ann Surg.

[30] Mendes OM Avaliação formativa no ensino superior: Reflexões e alternativas possíveis.

[31] Cosman P, Hemli JM, Ellis AM, Hugh T (2007). Learning the surgical craft a review of Skills training options. ANZ J Surg.

[32] Kolozsvari NO, Feldman LS, Vassiliou MC (2011). Sim one, do one, teach one Considerations in designing training curricula for surgical simulation. J Surg Educ.

[33] CFM OFÍCIO Nº. 0411/2022/DECCT/CFM.

